# Variations and Challenges in the Management of Traumatic Bladder Injuries: An Experience From a Large Trauma Center

**DOI:** 10.7759/cureus.82245

**Published:** 2025-04-14

**Authors:** Anna Nguyen, Se Jong Choi, Beshoy Gabriel, Stephen Chai, Jan Serrano, Fanglong Dong, Benjamin Archambeau, Michael M Neeki

**Affiliations:** 1 Department of Medicine, California University of Science and Medicine, Colton, USA; 2 Department of Emergency Medicine, Arrowhead Regional Medical Center, Colton, USA; 3 Department of Emergency Medicine, California University of Science and Medicine, Colton, USA; 4 Department of Orthopedic Surgery, California University of Science and Medicine, Colton, USA; 5 Department of Pediatrics, California University of Science and Medicine, Colton, USA; 6 Department of Trauma Surgery, Arrowhead Regional Medical Center, Colton, USA

**Keywords:** bladder rupture, bladder trauma, catheter drainage, conservative management, mechanism of injury

## Abstract

Introduction: Bladder trauma, often resulting from blunt or penetrating injuries, presents a significant challenge in trauma care due to its potential for serious complications and mortality. This retrospective study aimed to investigate demographic characteristics, mechanisms of injury, management strategies, and outcomes in bladder trauma patients, with a focus on comparing the differences between blunt and penetrating trauma cases.

Methods: Data were collected from trauma patients presenting to Arrowhead Regional Medical Center between February 17, 2013, and October 30, 2022. Patients aged 18 years and older diagnosed with bladder rupture were included in the final analysis. Patient demographics, mechanisms of injury, and treatment modalities were analyzed to compare the differences in outcomes. Statistical analyses were conducted using the Statistical Analysis System software for Windows version 9.4 (SAS Inc., Cary, NC).

Results: A total of 88 patients were included in the analysis. The majority of patients were men (n = 65, 73.9%), and blunt trauma accounted for 75% (n = 66) of cases. While there was a significantly higher proportion of men in penetrating trauma cases, differences in mortality, systolic and diastolic blood pressure, pulse, respiratory rate, oxygen saturation, Glasgow Coma Scale, injury severity score, and units of blood given were not statistically different between blunt and penetrating trauma cases.

Conclusion: There is no statistical difference in regard to outcomes between blunt and penetrating bladder trauma. Future research should aim to validate these findings in larger cohorts, explore additional factors influencing treatment decisions and outcomes, and investigate optimal management strategies to enhance patient care and minimize complications in this challenging clinical scenario.

## Introduction

Bladder trauma ranks as the second most common genitourinary injury, often occurring in cases of blunt trauma to the abdomen and pelvis [1]. The majority of bladder ruptures, between 80% and 90%, are extraperitoneal, usually as a result of pelvic fractures caused by severe force to the anterior bladder/abdomen [2]. Although less common, the bladder can also be injured from iatrogenic causes, with incidence being highest in gynecologic and urologic surgeries [3]. In contrast, intraperitoneal bladder ruptures usually occur because of a rise in intravesical pressure following trauma. The bladder becomes full and protrudes over the pelvic bone, leading to rupture of a weak point, typically the dome [2].

The management of traumatic bladder injuries differs significantly between blunt and penetrating trauma. Blunt trauma, which accounts for 60%-85% of bladder injuries, is most commonly associated with motor vehicle accidents and pelvic fractures [4]. In these cases, extraperitoneal injuries are more frequent and can often be managed conservatively with catheter drainage [5]. Conversely, penetrating trauma, such as gunshot wounds or stab injuries, is more likely to result in intraperitoneal bladder rupture, which typically requires surgical intervention [2,6]. The literature suggests that while uncomplicated extraperitoneal injuries from blunt trauma may be successfully treated with catheter drainage, complex extraperitoneal injuries or those associated with other abdominal injuries may benefit from surgical interventions [7]. Intraperitoneal injuries, regardless of the mechanism, generally necessitate operative management to prevent complications such as peritonitis and sepsis [6]. Of note, Mahat et al. described successful conservative management of small intraperitoneal ruptures in select patients with close monitoring and prolonged catheter drainage [2].

The variations in management approaches highlight the importance of continued research in current clinical practices. This study aims to investigate the demographic characteristics, mechanisms of injury, management, and treatment outcomes of bladder trauma patients, with a focus on comparing the differences between blunt and penetrating trauma cases. Our research contributes valuable data to the existing body of knowledge on traumatic bladder injuries to inform future clinical practices and patient care strategies.

## Materials and methods

Study design

This retrospective study analyzed consecutive patients who presented to the Arrowhead Regional Medical Center (ARMC) for trauma care with suspected bladder rupture between February 17, 2013, and October 30, 2022. ARMC is a 456-bed acute care teaching facility and an American College of Surgeons-certified level I trauma center in San Bernardino County, California. The ARMC emergency department is one of the busiest in the state of California, with more than 100,000 visits and over 3,000 adult trauma cases annually [8].

Study population and ethics statement

Patients 18 years and older were included in this study. Patients diagnosed with bladder rupture as the primary diagnosis at admission and discharge were identified using the International Classification of Diseases, Ninth and Tenth Revision Code [9,10]. Patient demographics were abstracted from their electronic medical records, including race, marital status, and insurance status. This study was approved by the institutional review board at ARMC with the approval number 23-06. Informed consent was waived, and data were reported in an aggregated format. No patients were identified in this manuscript.

Statistical analysis

All analyses were conducted using the Statistical Analysis System software for Windows version 9.4 (SAS Inc., Cary, NC). Descriptive statistics were presented as follows: mean ± standard deviation or median and first and third quartiles for continuous variables and frequency and percentage for categorical variables. Chi-square crosstab analyses were conducted to assess the association between categorical variables and the mechanism of injury (blunt vs. penetrating). If the expected cell count is less than five, Fisher’s exact test will be used. Independent t-tests were conducted to assess the mean of continuous variables between the two types of mechanisms of injury. Wilcoxon rank-sum tests were conducted to assess the median of continuous variables between the two types of mechanisms of injury. All statistical analyses were two-sided. A p value of <0.05 was considered to be statistically significant.

## Results

A total of 88 patients were included in the final analysis. More than half (73.9%, n = 65) of patients were men, the average age was 33.84 years, and 75% (n = 66) sustained blunt trauma.

Table 1 presents the comparison results between blunt and penetrating trauma. There was a higher percentage of men in the penetrating trauma compared to the blunt trauma (90.9% vs. 68.2%, p = 0.0356). Though not statistically significant, a higher percentage of blunt injury than penetrating injury was managed using nonsurgical methods (34.9% vs. 13.6%, p = 0.059). No statistical significance was detected in other variables between blunt vs. penetrating trauma.

**Table 1:** Comparison of clinical outcomes between blunt and penetrating trauma All values were presented as mean ± standard deviations (SD), median with first and third quartiles inside the parenthesis, or frequency with corresponding column percentage inside the parenthesis ^*^p value was calculated through Fisher’s exact test SD: standard deviation

Parameters	Blunt (n = 66)	Penetrating (n = 22)	p value
Gender, n (%)			
Female	21 (31.8%)	2 (9.1%)	0.0356
Male	45 (68.2%)	20 (90.9%)	
Management of bladder injury, n (%)			
Nonsurgical	23 (34.9%)	3 (13.6%)	0.059
Surgical	43 (65.2%)	19 (86.4%)	
Death, n (%)	5 (7.6%)	0 (0%)	0.3254^*^
Age, mean ± SD	35.32 ± 15.5	29.41 ± 10.76	0.101
Systolic blood pressure, mean ± SD	126.17 ± 28.38	139.48 ± 21.1	0.051
Diastolic blood pressure, mean ± SD	82.65 ± 24.36	87.81 ± 22.33	0.392
Pulse, mean ± SD	95.26 ± 23.1	99.5 ± 27.9	0.483
Respiratory rate, mean ± SD	20.3 ± 4.98	20.24 ± 3.88	0.959
Oxygen saturation, mean ± SD	96.98 ± 5.46	98.14 ± 2.41	0.181
Glasgow Coma Scale, median (Q1, Q3)	15 (14, 15)	15 (15, 15)	0.226
Injury severity score. median (Q1, Q3)	17 (14, 22)	10 (9, 25)	0.163
Amount of blood unit, median (Q1, Q3)	1 (0, 5)	1 (0, 5)	0.157

## Discussion

This study noted the blunt trauma cases as the most common mechanism of traumatic bladder injury (75%, n = 66), which is in agreement with the existing literature [2]. More than half (73.9%, n = 65) of patients were men, a fact especially pronounced in cases of penetrating trauma compared to blunt trauma (90.9% vs. 68.2%, p = 0.0356). The observed gender disparity in traumatic bladder injuries, with a higher proportion of male patients, especially in the cases of penetrating trauma, highlights the potential influence of gender-specific factors on injury patterns and outcomes. Men have been shown to engage more frequently in risky behaviors, including reckless driving, which was the most common mechanism of blunt trauma and violence, and firearms, which was the most common mechanism of penetrating trauma [5].

As expected, blunt injury was managed using nonsurgical methods more frequently than penetrating injury (34.9% vs. 13.6%, p = 0.059), though this difference did not reach statistical significance. Nonsurgical interventions may be preferred for blunt trauma due to the extraperitoneal nature of most injuries. In these cases, the tear usually occurs through the serosal layer of the bladder wall, allowing urine to leak into the extraperitoneal space [11]. The peritoneum, the thin membrane lining the abdominal cavity, remains intact, preventing urine from entering inside the peritoneum, allowing for conservative management with catheterization to be sufficient for healing, as the risk of infection is low [1]. Draining urine from the bladder helps relieve pressure, preventing potentially harmful leakage through the rupture. More complex injuries, however, call for surgical repair to prevent severe complications such as peritonitis and sepsis.

Diagnostic recommendations by the American Urological Association emphasize the use of retrograde cystography or computed tomography in stable patients with gross hematuria and pelvic fractures. Pelvic fractures alone do not call for bladder evaluation, but there has been a significant association with gross hematuria in combination with pelvic fractures as a sign of bladder injury [12]. For stable patients with gross hematuria and indicators of bladder injury, or in cases of pelvic ring fractures showing signs of bladder rupture (e.g., suprapubic pain, bladder distention, difficulty or inability to void, and gross hematuria), retrograde cystography is also advised [6,13]. However, regardless of recommendations, physicians should use their best judgment and clinical assessment to determine the necessity of imaging to ensure accurate identification and appropriate management of bladder injuries.

Postoperative complications in traumatic bladder injuries can arise from various factors, one being prolonged catheterization [14]. Prolonged catheterization can lead to retrograde spread of bacteria from the urinary tract, increasing the risk of urinary tract infection and orchitis. It can also contribute to persistent hematuria, surgical site infection, and urinary leakage. Additionally, catheter-related trauma may cause local tissue damage and delayed wound healing, which may predispose patients to abnormal fistulas between the bladder and other surrounding structures [4,15]. Future research should focus on identifying optimal catheterization durations and alternative management strategies to reduce the risk of such adverse outcomes.

The study also found a nonstatistically significant increase in systolic blood pressure between penetrating and blunt trauma (139.48 ± 21.1 vs. 126.17 ± 28.38, p = 0.051). This difference may be attributed to the intense pain caused by the direct injury to the bladder, triggering the sympathetic nervous system to raise the systolic blood pressure [16]. Thus, the pattern of systolic blood pressure can vary depending on the mechanism and severity of the trauma, highlighting the importance of comprehensive assessment and a tailored approach to care.

Finally, no statistical significance was detected on other variables between blunt and penetrating trauma, as shown in Table 1. In particular, the lack of a significant difference in mortality rates between blunt and penetrating trauma cases in this study indicates that, while the mechanism of injury might influence the management approach, the overall prognosis may depend more on the severity and the presence of concurrent injuries.

This study contains several limitations, including the small sample size and potential confounding factors, which highlight the need for larger, multicenter studies to further elucidate the optimal management strategies for traumatic bladder injuries. The small sample size in this study reduces the generalizability of the findings. Additionally, the complexity of trauma to the abdomen introduces confounding variables beyond bladder injury, potentially influencing observed outcomes.

## Conclusions

Overall, the diagnostic approaches seen in the cases studied aligned with standard management recommendations, including the use of retrograde cystography and CT. The higher prevalence of nonsurgical management in blunt trauma cases also aligns with current guidelines for the management of uncomplicated extraperitoneal injuries. However, the lack of significant differences in outcomes between blunt and penetrating trauma suggests that injury severity and associated injuries may be more critical determinants of prognosis than the mechanism itself. Future research should focus on identifying predictors of successful nonoperative management in both blunt and penetrating trauma, evaluating long-term outcomes and complications associated with different management approaches, and developing standardized protocols for the assessment and treatment of bladder trauma based on injury mechanism and severity.
